# Predictors of persistent stress urinary incontinence after transvaginal mesh repair

**DOI:** 10.1186/s12905-018-0667-0

**Published:** 2018-10-25

**Authors:** Shohei Kawaguchi, Kazutaka Narimoto, Satoko Urata, Masami Takeyama, Yoshifumi Kadono, Atsushi Mizokami

**Affiliations:** 1 Department of Integrative Cancer Therapy and Urology, Kanazawa University Graduate School of Medical Science, 13-1, Takaramachi, Kanazawa, Ishikawa 920-8641 Japan; 2 Department of Urogynecology, First Towakai Hospital, 2-17, Miyanochou, Takatsuki, Osaka, 569-0081 Japan

**Keywords:** Pelvic organ prolapse, Stress urinary incontinence, Transvaginal mesh, Mid-urethral sling, Voiding function

## Abstract

**Background:**

We evaluated the effect of transvaginal mesh (TVM) surgery for voiding function and continence using noninvasive examination and questionnaire. The present study aimed to ascertain which categories of patients need concomitant mid-urethral sling (MUS) after TVM surgery.

**Methods:**

We included women who underwent TVM procedure between November 2009 and October 2013. Data from noninstrumented uroflowmetry and questionnaires about urinary symptoms were analyzed.

**Results:**

The present study investigated the cases of 961 women who underwent TVM surgery. The persistence of stress urinary incontinence (SUI) was 57.6%. Almost all the parameters measured using uroflowmetry and questionnaires significantly improved in all types of urinary incontinence 12 months after surgery. A history of hysterectomy, preoperative high flow (corrected maximum flow rate > 1.5), and preoperative urge urinary incontinence were independent risk factors for the persistence of SUI.

**Conclusions:**

TVM for pelvic organ prolapse improved subjective and objective voiding function. Mixed urinary incontinence (MUI) patients with high urinary flow may be suitable for concomitant MUS with TVM because of the high level of SUI persistence.

## Background

Pelvic organ prolapse (POP) is frequent, with a prevalence that ranges from 2.9 to 11.4% when assessed by questionnaire and from 31.8 to 97.7% when evaluated by clinical examination [1]. The lifetime risk of undergoing surgery for POP is reported to be about 20% [2]. POP is often associated with lower urinary tract symptoms such as voiding dysfunction, urinary frequency, and incontinence. Voiding dysfunction may be attributed to bladder outlet obstruction because of mechanical urethral kinking, or urethral and/or bladder neck compression by the prolapsed cystocele [3]. Uterine prolapse or rectocele may also contribute to bladder outlet obstruction. Overactive bladder and POP are strongly correlated, and bladder outlet obstruction is likely the cause of this relationship [4]. Stress urinary incontinence (SUI) tends to be more prevalent in middle-aged women and commonly occurs with POP. Urinary incontinence is a serious problem among middle-aged women because it has a detrimental impact on health-related quality of life (QOL) [5].

The symptoms of voiding dysfunction and overactive bladder associated with POP are usually mitigated by POP repair (surgery or ring pessary) [6]. Our previous study showed that women with untreated POP possessed impaired detrusor contractility and bladder outlet obstruction, which significantly improved after transvaginal mesh surgery (TVM) [7]. However, the effect of pelvic reconstructive surgery on the prevalence of SUI is complicated. SUI may improve or worsen after POP surgery. A study has reported that 39% of the patients with preoperative SUI were cured after POP surgery. However, de novo SUI appeared in 22% of the patients without preoperative SUI [8].

In women with POP and coexisting SUI, concomitant mid-urethral sling (MUS) with POP surgery results in a high cure rate of SUI at 1-year follow-up (success rate 95%) [9]. However, complications increase in patients who undergo concomitant MUS compared to those who undergo POP repair alone. Prophylactic MUS for patients without preoperative SUI reduces the likelihood of urinary incontinence but increases the likelihood of adverse events [10]. The number of individuals that needs to be treated using a sling to prevent one case of urinary incontinence is 6.3 in patients without preoperative SUI, and this is unsatisfactory. Guidelines indicate that women may be offered the choice of not treating evident SUI at the same time as prolapse surgery, as long as they are warned that a second intervention may be required later [1]. Patients need more information to decide whether to undergo concomitant MUS given that this procedure for priventing incontinence could be unnecessary.

In addition, voiding dysfunction following anti-incontinence surgery is not uncommon. Although MUS produces a dynamic urethral kinking without compressing the urethra at rest, 4–43% of patients develop obstructive voiding symptoms. Han et al. showed that maximum flow rate is significantly reduced at long-term follow-up after MUS [11]. Therefore, MUS may be unsuitable for patients with low urinary flow. Accordingly, when concomitant MUS with POP surgery is elected, it is important to predict low urinary flow after POP surgery.

In this study, we evaluated the effect of TVM surgery on voiding function and continence using noninvasive examination and questionnaire. In addition, we considered which categories of patients need concomitant MUS with TVM surgery.

## Methods

The study population comprised women who underwent TVM surgery for POP at the urogynecology center of Senboku-Fujii Hospital between November 2009 and October 2013. The surgical procedure using soft polypropylene mesh was based on that reported by the French TVM group (Gynemesh PS™) [12]. The mesh was cut into a shape similar to that of Prolift™ as previously reported [13] [14]. Staging of POP was performed using a pelvic organ prolapse quantification assessment (POP-Q). The cystocele stage was assessed by point Ba and the uterine or vault prolapse stage was assessed by point C. Patients with cystoceles underwent anterior TVM only, whereas those requiring level 1 or 2 repair for conditions including uterine prolapse or vault prolapse, were treated by anterior and posterior TVM surgery. Patients were excluded from the study if they met the following criteria: they had undergone any previous anti-incontinence surgery; they did not report dates of the pre- or postoperative uroflowmetry, their voided volumes were less than 50 ml; or they did not complete the questionnaires. The present study was approved by the Institutional Ethics Committee of the Senboku-Fujii Hospital. The research content is posted on outpatient of Senboku-Fujii Hospital after approval and data of cases without opt-out intention were adopted.

Measurements were performed before surgery and at 12 months follow-up. Physical examination including POP-Q was performed. Noninstrumented uroflowmetry and measurement of post-voided residual urine was performed using ultrasonography. Patients were asked to void with a comfortably full bladder, in sitting position, into a gravimetric uroflowmeter. Based on the guideline [15], the maximum flow rate (Qmax), average flow rate (Qave), voided volume (VV), post-void residual volume (PVR) were recorded. Corrected Qmax (cQmax) and Qave (cQave) were calculated using the formulae Qmax/$\sqrt{VV}$ and Qave/$\sqrt{VV}$ respectively [16, 17].

Data regarding Overactive Bladder Symptom Score (OABSS), International Prostate Symptom Score and QOL score (IPSS/QOL), and International Consultation on Incontinence Questionnaire-Short Form (ICIQ-SF) were acquired from the questionnaires. Patients were categorized into groups according to their category of urinary incontinence (UI) (patients without UI: No UI Group; patients with pure SUI: SUI Group; patient with pure urge urinary incontinence: UUI Group; and patients with mixed urinary incontinence: MUI Group). UI was classified on the basis of self-diagnostic items of ICIQ-SF. SUI was diagnosed if the patient experienced urine leakage during coughing, sneezing, or physical activity; UUI was diagnosed if the patient experienced urine leakage before she could reach the toilet; and MUI was diagnosed if the patient had both SUI and UUI as Abdullah et al. previously reported [18].

The primary outcome of this study was the persistence of SUI in patients with preoperative SUI. Further, this study measured the occurrence of de novo SUI and UUI, and the persistence of UUI. We compared SUI persistence in patients who had preoperative pure SUI with those who had preoperative MUI using the chi-square test. UUI persistence was similarly compared. Changes recorded by the uroflowmetry and questionnaire responses were evaluated. Comparisons of the uroflowmetric data and questionnaire replies before and 12 months after TVM surgery were performed using the Wilcoxon signed-rank test. Univariable logistic regression was used to identify risk factors for postoperative low urinary flow and SUI persistence. The considered risk factors were patient age, parity, body mass index (BMI), the presence of diabetes mellitus, the presence of cerebrovascular disease, previous hysterectomy, POP stage, cQmax, and preoperative UUI. Odds ratios (OR) and 95% confidence interval (CI) for postoperative low urinary flow and SUI persistence are presented. Multivariable logistic regression was performed to identify independence of variables. Variables with a *P*-value < 0.1 in the univariable analysis were included in multivariable analysis. In all analyses, *p* < 0.05 indicates statistical significance.

## Results

The present study investigated the cases of 961 women who underwent TVM surgery. Table 1 shows the characteristics of the women included in the study. Nineteen patients experienced intraoperative complications involving bladder perforation and 5 patients experienced more than 300 ml blood loss. Postoperative complication were graded using the Clavien–Dindo classification and the grade 2 complication rate was 6.9% (residual urine/ urinary retention [*n* = 60], hematoma [*n* = 5], pelvic abscess [*n* = 1]). No patients experienced greater than grade 2 complications.

**Table 1:** Patient characteristics

Characteristic	*N* = 961
Age (years)	68 (43–89)
Parity (n)	2 (0–9)
Body mass index (kg/m^2^)	23.5 (16.4–35.1)
Postmenopausal states (yes)	939 (97.7%)
Pelvic organ prolapse	
Stage 2	91 (9.5%)
Stage 3 or 4	870 (90.5%)
Surgical site	
Anterior compartment	930 (96.8%)
Posterior compartment	519 (54.0%)
Previous pelvic organ prolapse surgery	60 (6.2%)
Previous hysterectomy	150 (15.6%)
Diabetes mellitus	88 (9.2%)
Cerebrovascular disease	38 (4.0%)
Anatomical recurrence at 1-year (POP-Q > Stage 1)	16 (1.7%)

*POP-Q* pelvic organ prolapse quantification assessment

Eighteen patients (1.9%) underwent concomitant MUS, and 34 patients (3.5%) underwent postoperative MUS before 12 months follow-up. Changes in the incidence of incontinence before and after TVM surgery were evaluated; patients who underwent MUS after 12 months follow-up were excluded (Table 2). SUI persistence was 57.6% (258/448) and UUI persistence was 37.4% (134/358). De novo SUI rate was 36.6% (161/440) and de novo UUI rate was 8.3% (44/530). SUI persistence in preoperative MUI patients was 63.4% (121/191) and was significantly higher than that in preoperative pure SUI patients (53.3% 137/257 *p* = 0.033). There was no significant difference between the UUI persistence in preoperative MUI patients (40.3% 77/191) and that on preoperative pure UUI patients (34.1% 57/167 *p* = 0.228).

**Table 2:** Changes in the types of incontinence before and after surgery

	Preoperative			
	No UI	SUI	UUI	MUI
	*N* = 273 (%)	*N* = 257 (%)	*N* = 167 (%)	*N* = 191 (%)
Postoperative				
No UI	173 (63.4)	113(44.0)	64 (38.3)	43 (22.5)
SUI	79 (28.9)	118 (45.9)	44 (26.3)	68 (35.6)
UUI	10 (3.7)	5 (1.9)	29 (17.4)	24 (12.6)
MUI	10 (3.7)	19 (7.4)	28 (16.8)	53 (27.7)
Unclassified	1 (0.4)	2 (0.8)	2 (1.2)	3 (1.6)

*No UI* no urinary incontinence, *SUI* stress urinary incontinence, *UUI* urge urinary incontinence, *MUI* mixed urinary incontinence

The number of patients with preoperative unclassified UI is 21

Table 3 shows the changes in uroflowmetric parameters and responses to questionnaires about urinary symptoms. All the parameters of uroflowmetry significantly improved in all types of UI 12 months after surgery. In questionnaires about urinary symptoms, the responses, apart from the ICIQ-SF score of patients without preoperative UI, showed significant improvement regardless of the types of UI 12 months after surgery.

**Table 3:** Changes in uroflowmetry parameters and results of questionnaires on urinary symptoms before and 12 months after surgery

	No UI			SUI			UUI			MUI		
	pre	post	*p*	pre	post	*p*	pre	post	*p*	pre	post	*p*
cQmax	1.16 ± 0.46	1.46 ± 0.61	0.000	1.22 ± 0.49	1.53 ± 0.58	0.000	1.11 ± 0.44	1.43 ± 0.59	0.000	1.21 ± 0.53	1.57 ± 0.68	0.000
cQave	0.61 ± 0.27	0.91 ± 0.37	0.000	0.64 ± 0.27	0.96 ± 0.38	0.000	0.55 ± 0.23	0.89 ± 0.36	0.000	0.63 ± 0.29	0.97 ± 0.40	0.000
PVR (mL)	18.0 ± 52.5	8.6 ± 29.5	0.015	17.5 ± 55.2	5.8 ± 25.8	0.002	38.4 ± 79.4	7.8 ± 26.1	0.000	19.1 ± 5.38	6.0 ± 25.6	0.002
OABSS	2.8 ± 2.2	2.3 ± 1.8	0.001	3.2 ± 2.7	2.7 ± 2.3	0.000	6.3 ± 3.1	4.1 ± 3.0	0.000	6.6 ± 3.4	4.1 ± 3.0	0.000
IPSS	8.0 ± 7.0	4.5 ± 4.8	0.000	9.0 ± 7.2	4.5 ± 4.7	0.000	11.5 ± 7.0	6.3 ± 9.0	0.000	13.1 ± 8.9	5.5 ± 5.1	0.000
ICIQ-SF	2.7 ± 2.9	2.6 ± 3.1	0.737	7.4 ± 3.3	4.5 ± 4.2	0.000	9.2 ± 3.8	4.9 ± 4.0	0.000	11.0 ± 4.5	5.9 ± 4.4	0.000
QOL score	3.9 ± 1.7	1.7 ± 1.3	0.000	4.5 ± 1.5	2.0 ± 1.5	0.000	5.1 ± 1.2	2.3 ± 1.6	0.000	5.3 ± 1.0	2.3 ± 1.6	0.000

*cQmax* Corrected maximum flow rate, *cQave* Corrected average flow rate, *PVR* Post-void residual urine, *OABSS* Overactive bladder symptom score, *IPSS* International prostate symptom score, *ICIQ-SF* International consultation on incontinence questionnaire – Short Form

Table 4 shows the OR for the risk of SUI persistence using univariable and multivariable logistic regression. This study identified a history of hysterectomy, preoperative high flow (cQmax > 1.5), and preoperative UUI as independent risk factors for SUI persistence (*p* < 0.05). Table 5 presents the univariable and multivariable analysis of postoperative low urinary flow (cQmax < 1.0). This study identified the presence of diabetes mellitus and preoperative low urinary flow (cQmax < 1.0) as independent risk factors for postoperative low urinary flow (*p* < 0.05).

**Table 4:** Odds ratios for the risk of postoperative stress urinary incontinence using univariable and multivariable logistic regression

Factor	Patients with preoperative SUI			
	Univariable logistic regression		Multivariable logistic regression	
	*p*-value	OR (95% CI)	*p*-value	OR (95% CI)
Age (> 68 years old)	0.716	1.073 (0.734–1.570)		
Parity (> 3 times)	0.082	1.436 (0.956–2.159)	0.060	1.492 (0.983–2.264)
BMI (> 24 kg/m^2^)	0.778	1.056 (0.722–1.545)		
Diabetes mellitus	0.320	1.426 (0.709–2.867)		
Cerebrovascular disease	0.924	0.958 (0.395–2.322)		
Hysterectomy history	0.065	1.704 (0.967–3.002)	0.046	1.802 (1.010–3.217)
POP stage (> Stage 3)	0.276	1.436 (0.749–2.754)		
cQmax (> 1.5)	0.003	2.050 (1.274–3.299)	0.002	2.147 (1.325–3.480)
UUI preoperatively	0.028	1.543 (1.047–2.272)	0.036	1.525 (1.027–2.269)

*BMI* Body mass index, *POP* Pelvic organ prolapse, *cQmax* Corrected maximum flow rate, *UUI* Urge urinary incontinence

**Table 5:** Odds ratios for the risk of postoperative low urinary flow (cQmax < 1.0) using univariable and multivariable logistic regression

Factor	Patients with preoperative SUI			
	Univariable logistic regression		Multivariable logistic regression	
	*p*-value	OR (95% CI)	*p*-value	OR (95% CI)
Age (> 68 years old)	0.086	1.585 (0.937–2.682)	0.511	1.206 (0.690–2.107)
Parity (> 3 times)	0.989	1.004 (0.576–1.749)		
BMI (> 24 kg/m^2^)	0.237	0.723 (0.423–1.237)		
Diabetes mellitus	0.001	3.444 (1.660–7.145)	0.004	3.112 (1.435–6.748)
Cerebrovascular disease	0.481	0.587 (0.134–2.582)		
Hysterectomy history	0.356	1.385 (0.694–2.762)		
POP stage (> Stage 3)	0.164	0.569 (0.257–1.258)		
cQmax (< 1.0)	0.000	3.431 (1.989–5.918)	0.000	3.286 (1.885–5.728)
UUI preoperatively	0.575	1.162 (0.687–1.967)		

*BMI* Body mass index, *POP* Pelvic organ prolapse, *cQmax* Corrected maximum flow rate, *UUI* Urge urinary incontinence

## Discussion

Various surgical procedures for POP have been developed to date. Traditional colporrhaphy for POP using native tissue has a high anatomic failure rate of 58% [19]. Compared with native tissue repair, TVM surgery yields better anatomic results [20]. The study reported the anatomical success rate of Prolift™ was 87%. However, it is also reported that complications increase because of TVM surgery. As compared with anterior colporrhaphy, TVM surgery for cystocele repair has a high success rate but also a high complication rate [21]. Food and Drug Administration has announced that serious complications associated with TVM procedure are not uncommon. In recent years, laparoscopic or robotic sacral colpopexy has been considered to be the “gold standard” for POP surgery because of fewer mesh-related complications [22]. However, sacral colpopexy is more time-consuming and expensive than native tissue repair or TVM surgery. In addition, the degree of difficulty of sacral colpopexy may increase because of intraperitoneal adhesion caused by a previous lower abdominal operation, obesity and other factors. It is reported that TVM surgery may provide the benefits of low complication rates and a QOL comparable to that of postmenopausal women [23]. TVM can be effective and safe depending on appropriate patient selection and advanced surgical skill.

Previously, we have shown that women with untreated POP have impaired detrusor contractility and bladder outlet obstruction, which significantly improve after TVM surgery [7]. The present study also showed that TVM surgery improved both objective and subjective urinary function. It is reported that approximately 40% of women with symptoms of UUI or SUI were cured by POP surgery [8]. Similarly, the present study showed that 41% of women with preoperative SUI were cured by TVM surgery alone. However, de novo SUI appeared in 36% of women who underwent TVM surgery, which is an important drawback of POP surgery because of its impact on QOL.

The previous report showed that concomitant MUS with POP surgery reduced postoperative SUI in women with preoperative SUI [9]. It is also reported that a prophylactic concomitant MUS with POP surgery prevented postoperative SUI in women without SUI [10]. However, concomitant MUS increases adverse events such as hematoma, bladder perforation, urethral mesh erosion, severe infection, and voiding dysfunction [9, 10]. Approximately 40% of patients with preoperative SUI cured by POP surgery alone, and at least 60% of patients without preoperative SUI do not develop de novo SUI. To prevent unnecessary surgery, to careful assessment is essential before performing concomitant MUS.

Some studies have used multichannel urodynamic testing to identify the predictors of postoperative UI. Our previous investigation showed that urethral obstruction was an independent predictor of de novo SUI [24]. In addition, age (> 66 years), the presence of diabetes mellitus, preoperative low maximum urethral closure pressure (< 60 mmH_2_O) and a functional urethral length of less than 2 cm were reported as the predictors of de novo SUI [25]. However, it may be difficult to achieve accurate and reproducible results of a POP patient’s multichannel urodynamic study because the results may differ according to the method and degree of POP reduction at examination.

There were limitations to this retrospective study. Firstly, a considerable number of patients were excluded because of incomplete data. Exclusion of incomplete data meant there was a possibility that selection bias had been applied. Secondly, the classification of UI may not be accurate enough because we classified types of UI based on subjective data. However, the present study identified previous hysterectomy, preoperative high flow, and preoperative UUI as independent risk factors for SUI persistence. It is reported that surgical treatment of MUI with MUS results in cure or improvement of urinary urgency, frequency and UUI in 30–70%, SUI in approximately 80–90% patients [26]. This study showed that the SUI persistence in preoperative MUI patients was significantly higher than that in preoperative pure SUI patients. That indicated that preoperative UUI was a factor contributing to postoperative persistence of SUI. SUI persistence in patients with MUI and high urinary flow (cQmax > 1.5) was high 75.0% (33/44), compared with the total of patients with SUI and MUI 57.5%, (258/448). Patients with MUI and high urinary flow may be candidates for concomitant MUS with TVM surgery, given that three quarters of patients may benefit from concomitant surgery. This study also identified the presence of diabetes mellitus and preoperative low urinary flow as independent risk factors for postoperative low urinary flow. In our previous study, we demonstrated that Qmax and Qave in uroflowmetry decreased after MUS [17]. Not performing concomitant MUS with POP surgery for patients with diabetes mellitus and preoperative low urinary flow may be feasible.

## Conclusions

TVM for pelvic organ prolapse improved subjective and objective voiding function. This study showed that preoperative high flows, MUI, and a history of hysterectomy predicted persistent SUI. Patients with MUI and high urinary flow may be candidates for concomitant MUS with TVM because of the high incidence of SUI persistence.

## Abbreviations

cQave: corrected Qave
cQmax: corrected Qmax
MUI: mixed urinary incontinence
MUS: mid-urethral sling
POP: pelvic organ prolapse
PVR: post-void residual volume
SUI: stress urinary incontinence
TVM: transvaginal mesh surgery
UUI: urge urinary incontinence
VV: voided volume
